# Investigation of the Effect of Dehydroepiandrosterone (DHEA) on In Vitro Fertilization (IVF) Outcomes in a Patient With Poor Ovarian Reserve: A Case Report

**DOI:** 10.7759/cureus.63491

**Published:** 2024-06-30

**Authors:** Rupali Rao, Akash More, Jarul Shrivastava

**Affiliations:** 1 Clinical Embryology, Datta Meghe Institute of Higher Education and Research, Wardha, IND

**Keywords:** dhea, intrauterine cytoplasmic sperm injection insemination, clinical pregnancy, metroplasty, secondary infertility

## Abstract

This study represents a case of a 42-year-old female patient who had a history of infertility, undergoing treatment at the in vitro fertilization (IVF) centre in Sawangi, India. The patient presented with a medical history marked by recurrent medical termination of pregnancies, a small uterus diagnosed through metroplasty, and a significant impediment to fertility treatment attributed to poor ovarian reserve. Clinical assessment revealed the male partner's history of alcohol consumption and cigarette smoking, along with benazepril usage for hypertension (5 mg/day). Despite normal semen parameters, intrauterine insemination (IUI) proved unsuccessful, prompting a recommendation for IVF utilizing the intracytoplasmic sperm injection (ICSI) procedure. The patient displayed low anti-Mullerian hormone (AMH) levels, indicative of insufficient ovarian reserve. Dehydroepiandrosterone (DHEA) supplementation was advised orally for a period of one month to enhance ovarian function. Subsequent evaluation demonstrated a notable increase in AMH levels, facilitating the retrieval of six oocytes, comprising average-quality metaphase II (MII) oocytes and one dysmorphic metaphase I (MI) oocyte. Following ICSI, successful fresh embryo transfer ensued, resulting in a positive beta-human chorionic gonadotropin (β-hCG) test with serum β-hCG levels measuring 1676 mIU/mL, confirming the successful implantation of one of the two transplanted embryos. This case underscores the significance of DHEA supplementation in augmenting ovarian reserve and achieving favorable IVF-ICSI outcomes in patients with primary infertility. The holistic approach, encompassing lifestyle modifications and tailored medication, contributed to a positive pregnancy outcome. Further research is warranted to explore the broader implications of DHEA therapy in the management of infertility.

## Introduction

The condition characterized by the inability to achieve pregnancy following a year of consistent, unprotected sexual activity during the fertile phase of the menstrual cycle is termed infertility [1]. Notably, the past decade has witnessed significant progress and achievements across all facets of assisted reproduction. Although research on assisted reproductive technology (ART) historically emphasized female infertility, particularly in women beyond childbearing age, advancements have been multifaceted [2].

While gamete and embryo manipulation remain integral to infertility interventions, the success rate of in vitro fertilization (IVF) cycles leading to live births is approximately one-third in most infertility clinics. A substantial number of patients experience repeated unsuccessful attempts at conception [3]. A particularly challenging subset of cases involves poor ovarian responders, where ovarian aspiration of oocytes becomes a significant hurdle [2].

A critical clinical concern within this spectrum is poor ovarian reserve (POR), denoting insufficient ovarian contents even after ovarian stimulation in intracytoplasmic sperm injection (ICSI) or IVF cycles [4].

Dehydroepiandrosterone (DHEA) and its sulfated metabolite, DHEA-S, are produced and eliminated by the adrenal cortex's zona reticularis in response to adrenocorticotropin hormone [5]. While primarily synthesized by the adrenal gland, DHEA is also produced in smaller quantities by various tissues. Serving as a precursor to testosterone and estrogen, DHEA can undergo conversion to these hormones in multiple tissues, including the ovaries, testes, skin, and hair follicles [6,7]. This case report details the positive clinical pregnancy outcome in a quadragenarian female patient with a history of failed IVF treatment following the administration of DHEA for the management of her POR condition.

## Case presentation

Patient medical history

A 42-year-old quadragenarian female patient and her 49-year-old husband visited an IVF medical facility in Sawangi, India, seeking assistance for their infertility condition. The patient exhibited a regular menstrual cycle with an average 30-day frequency and a four-day duration of menstrual bleeding. The patient's medical history also revealed a series of medical termination of pregnancies (MTPs) or abortions. The initial MTP occurred one and a half months after six years of marriage, followed by a subsequent termination at the same gestational age after eight years of marital union.

The patient's husband was under a regimen of orally administered benazepril at 10 mg/day for hypertension management. There was no family history of asthma, tuberculosis, or thyroid gland disease. Semen analysis demonstrated normal parameters, including a sperm count of 80 million/mL and motility at 75%. The patient had a history of two unsuccessful attempts of intrauterine insemination (IUI) using the husband's semen. The investigation into the etiology of infertility underscored the potential involvement of the adrenal cortex's zona reticularis in the synthesis and clearance of the endogenous hormone DHEA and its active metabolite, DHEA-S. This regulatory process is under the influence of the adrenocorticotropin hormone. Given the complex medical scenario, the female patient received a recommendation for ICSI as part of the ART intervention. This decision corresponds to the evaluation of her health condition and the difficulties observed in successfully getting pregnant using traditional reproductive techniques

Clinical findings

The patient was diagnosed with primary infertility, marked by a follicle-stimulating hormone (FSH) level of 123.82 pg/mL. The female patient exhibited FSH, luteinizing hormone (LH), and anti-Mullerian hormone (AMH) concentrations of 7.9 mIU/mL, 0.34 mIU/mL, and 0.247 ng/mL, respectively. The diminished AMH level indicated POR. In the initial ovum aspiration procedure, a standard ovum aspiration needle size 4 was utilized to access follicles; however, no oocytes were retrieved due to insufficient ovarian reserve, linked to low AMH levels. The subsequent intervention involved the administration of DHEA to address ovarian reserve. A DHEA dosage of 50 mg was prescribed to elevate AMH levels for one month.

**Figure 1:**
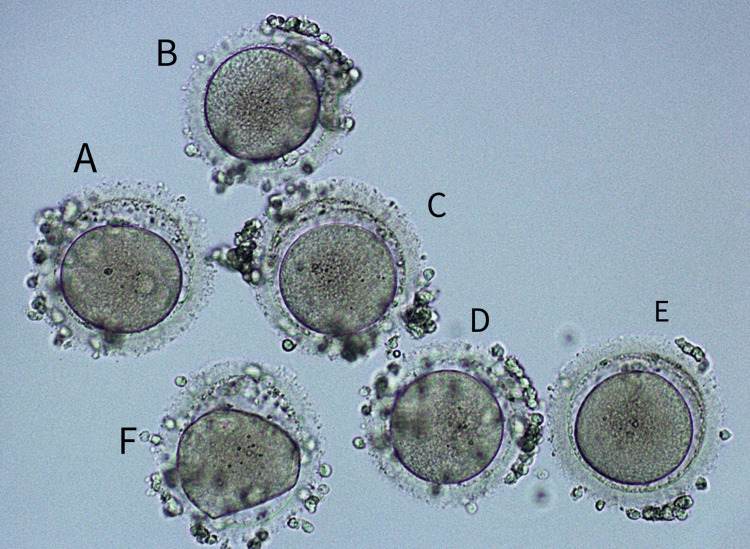
Six oocytes as retrieved from the patient A)-E): Average quality metaphase II oocytes; F) Dysmorphic metaphase I The original picture was clicked by the author of this article, and informed consent was obtained for publication from the patient

DHEA, a hormone produced by the adrenal gland, is sometimes utilized for its perceived benefits in enhancing sexual drive and overall well-being. The patient underwent a one-month DHEA treatment, with subsequent AMH and antral follicle count assessments aimed at gauging the impact of DHEA therapy on AMH levels. The results demonstrated a proportional increase in AMH levels with the duration of DHEA supplementation. Following this treatment, six oocytes were successfully retrieved and one dysmorphic metaphase I (MI) oocyte, as illustrated in Figures 1A-1F. ICSI was performed with the retrieved oocytes, with no complications reported during the ovum pick-up and ICSI procedures. A fresh embryo transfer was scheduled on day 21 post-ICSI, and the female patient experienced no complications following the embryo transfer procedure.

Follow-up

Upon successful completion of the procedure, with a subsequent recommendation for follow-up, the female patient was discharged. A prescription for oral ofloxacin tablets (200 mg) was provided, with instructions for morning and evening administration. Additionally, a daily regimen of vitamin B complex and iron supplements (400 mg) was advised, along with progesterone at a dosage of 500 mg per day.

Following 14 days of intralipid, hormonal support, and routine medical care, a blood sample was collected and subjected to a human chorionic gonadotropin (hCG) test. The test yielded a positive result, with the serum β-hCG levels measured at 1676 mIU/mL. Ultrasonography further confirmed the presence of a singleton pregnancy.

## Discussion

This case report serves as an illustrative instance of primary infertility treatment. Infertility secondary to reproductive tract infection stands as the predominant cause of female infertility [8].

DHEA medications are currently under evaluation for their impact on primary infertility outcomes in patients with POR [9]. While the use of DHEA is associated with an elevated pregnancy rate, our case study results indicate that the average oocyte retrieval is not significantly affected by its administration. Notably, DHEA usage may influence factors such as endometrial thickness or oocyte quality. Our findings reveal an improved prognosis for women afflicted with POR who undergo DHEA supplementation before ovarian stimulation, emphasizing the potential role of DHEA in the medical management of POR patients [10].

Consistent with earlier studies on IVF pregnancies by Ribbert et al. (1996), Heinonen et al. (1996), Frishman et al. (1997), and Wald et al. (1999) [11-14], our results indicate raised β-hCG levels in both IVF and ICSI pregnancies, with a more pronounced elevation in the IVF group. Antral follicular count (AFC) levels remained comparable to controls. However, our findings diverge from those reported by Lam et al. (1999), where ICSI pregnancies exhibited slightly lower β-hCG levels and significantly reduced AFC compared to controls [15].

By Jirge et al. (2014), our study aligns with the observation that women with POR, having experienced at least two prior failures attributed to POR, witness improvements in oocyte production, embryo quality, and live birth rates with DHEA supplementation [16]. Additionally, Zhang et al. (2023) reported increased clinical pregnancy rates, live birth rates, and ovarian reserve in poor ovarian response patients with DHEA supplementation [17]. Our current investigation contributes to the literature by demonstrating a significant increase in the number of retrieved oocytes, metaphase II (MII) oocytes, fertilized oocytes, day 3 embryos, and top-quality embryos at day 3 with DHEA pretreatment [18,19].

## Conclusions

In this particular case, the therapeutic application of DHEA was implemented to address the challenges of infertility, specifically focusing on the patient's insufficient ovarian reserve. The administration of DHEA supplements yielded positive outcomes, ultimately contributing to the successful initiation of pregnancy through IVF in the context of reproductive interventions conducted in Wardha, Maharashtra.

The intervention exhibited a notable enhancement in clinical pregnancies, indicating a promising trajectory in the management of POR with DHEA supplementation. However, it is crucial to underscore that this study's limited scope, confined to a singular patient, necessitates a cautious interpretation of the outcomes. Consequently, to establish robust and generalizable conclusions, further investigations employing a larger sample size are recommended. 
